# Spontaneous gram-negative bacillary meningitis in adult patients: characteristics and outcome

**DOI:** 10.1186/1471-2334-13-451

**Published:** 2013-09-30

**Authors:** Virginia Pomar, Natividad Benito, Joaquin López-Contreras, Pere Coll, Mercedes Gurguí, Pere Domingo

**Affiliations:** 1 Department of Internal Medicine, Infectious Diseases Unit, Hospital de la Santa Creu i Sant Pau, Institut d'Investigació Biomèdica Sant Pau, Universitat Autònoma de Barcelona, Spanish Network for Research in Infectious Diseases (REIPI), C/ Mas Casanovas 90, Barcelona 08025, Catalonia, Spain; 2 Department of Clinical Microbiology, Hospital de la Santa Creu i Sant Pau. Institut d'Investigació Biomèdica Sant Pau. Universitat Autònoma de Barcelona. Spanish Network for Research in Infectious Diseases (REIPI)., Barcelona, Spain

## Abstract

**Background:**

Spontaneous meningitis caused by gram-negative bacilli in adult patients is uncommon and poorly characterized. Our objective is to describe and compare the characteristics and the outcome of adult patients with spontaneous gram-negative bacilli meningitis (GNBM) and spontaneous meningitis due to other pathogens.

**Methods:**

Prospective single hospital-based observational cohort study conducted between 1982 and 2006 in a university tertiary hospital in Barcelona (Spain). The Main Outcome Measure: In-hospital mortality.

**Results:**

Gram-negative bacilli meningitis was diagnosed in 40 (7%) of 544 episodes of spontaneous acute bacterial meningitis. The most common pathogens were *Escherichia coli* and *Pseudomonas* species. On admission, characteristics associated with spontaneous gram-negative bacilli meningitis by multivariate modeling were advanced age, history of cancer, nosocomial acquisition of infection, urinary tract infection as distant focus of infection, absence of rash, hypotension, and a high cerebrospinal fluid white-cell count. Nine (23%) episodes were acquired in the hospital and they were most commonly caused by *Pseudomonas*. The in-hospital mortality rate was 53%. The mortality rate was higher among patients with Gram-negative bacillary meningitis than among those with other bacterial meningitis and their risk of death was twenty times higher than among patients infected with *Neisseria meningitidis* (odds ratio 20.47; 95% confidence interval 4.03-103.93; p<0.001).

**Conclusions:**

Gram-negative bacilli cause 9% of spontaneous bacterial meningitis of known etiology in adults. Characteristics associated with GNBM include advanced age, history of cancer, nosocomial acquisition, and urinary tract infection as distant focus of infection. The mortality rate is higher among patients with gram-negative bacillary meningitis than among those with other bacterial meningitides.

## Background

Bacterial meningitis is one of the main causes of infection-related death worldwide [1,2]. In developed countries, the estimated incidence is of 4–6 cases per 100,000 adults per year, being the most frequent etiologic agents in adults *Streptococcus pneumoniae* and *Neisseria meningitidis*[1-8].

Spontaneous meningitis caused by gram-negative bacilli (other than *Haemophilus influenzae*) in adults is an uncommon and poorly characterized disease. Most cases occur in neonates or infants and few studies have focused on the clinical features and prognostic factors of gram-negative bacillary meningitis (GNBM) in adults [7,9-20].

Much of the current understanding of GNBM in adults is based on studies that are limited by retrospective design and analysis that combine patients with spontaneous meningitis and meningitis secondary to trauma or neurosurgery [10-12,14-16]. However, gram-negative bacillary meningitis secondary to head and/or spinal trauma or neurosurgical procedures has specific characteristics that differentiate it from spontaneous gram-negative meningitis. Moreover, most of the scarce literature describing GNBM in adults was published more than a decade ago, and epidemiology and outcomes could have changed in recent years [9,10,12,14-20].

Using data from a large, prospective, single-hospital study of patients 14 years or older diagnosed with spontaneous bacterial meningitis over a 25-year period we intend to describe the characteristics spontaneous gram-negative bacillary meningitis in adults and to compare them with those of patients with spontaneous bacterial meningitis due to other pathogens.

## Methods

### Study population

We used data from a large, prospective, single-hospital cohort of patients with meningitis enrolled over a 25-year period at the Hospital de la Santa Creu i Sant Pau (Barcelona, Spain). This institution is a 620-bed tertiary university hospital providing care for an urban area with an estimated population of 450,000 inhabitants.

From 1982 through 2006 all consecutive adults (defined as patients 14 years or older) in whom acute bacterial meningitis was diagnosed at our hospital were prospectively identified.

The diagnosis of meningitis caused by a specific bacterial pathogen was based on compatible clinical findings (sudden onset of headache, fever, nausea, vomiting, neck stiffness and/or altered mental status) and one of the following: a positive cerebrospinal fluid (CSF) culture, or a negative CSF culture with a finding of neutrophilic pleocytosis (defined as pleocytosis of at least 100 neutrophils per cubic millimeter) and at least one of the following: a positive CSF antigen test, a positive blood culture, or identification of gram-negative diplococci on Gram’s staining of CSF in patients with a petechial or purpuric rash and a fulminant course (these last cases were considered to be caused by *Neisseria meningitidis*) [21]. In addition, episodes of "culture-negative" bacterial meningitis were included if patients had a compatible clinical picture along with neutrophilic pleocytosis and any of the following CSF abnormalities: depressed CSF glucose (defined as a CSF/blood glucose ratio <0.40) and elevated CSF protein (defined as >0.5g/l) [3,7,22]. Cases of viral, fungal, or mycobacterial meningitis were not included. Patients with history of recent (<6 months) neurosurgical procedures or traumatic head/spinal injury were excluded.

A standard case report form including demographic, clinical variables, laboratory and microbiologic results (isolates and susceptibility tests), complications, treatment and outcome was used to collect data.

The study and its subsequent amendments were approved by the Ethical Review Committee of the Hospital de la Santa Creu i Sant Pau. Written informed consent was obtained from the patient’s or their legal representatives.

### Patient selection

We included patients with spontaneous gram-negative bacillary meningitis. Patients were required to have a diagnosis of bacterial meningitis according to the previously mentioned criteria and the additional following criteria: isolation of gram-negative bacilli from the CSF or blood cultures. We excluded patients with *H.influenzae* meningitis (n = 13) from this study because it has specific and different characteristics and outcome.

### Microbiology methods

Isolates were obtained from routine cultures and were identified using standard methods [23]. The disc diffusion susceptibility test was performed according to Clinical Laboratory Standards Institute (CLSI) guidelines [24], using commercially available discs (Bio-Rad, Marnes La Coquette, France). MICs were determined using the broth micro dilution method according to the CLSI guidelines [25] using commercial panels (Sensitre, Trek diagnostic systems, West Sussex, England) or Etest (AB Biodisk, Solna, Sweden) according to the recommendations of the manufacturer.

### Definitions

Comorbidity was scored through the Charlson Comorbidity scale [26].

A primary distant focus of infection was considered when a patient had clinical symptoms and signs consistent with a focal infection distant from the central nervous system and the same pathogenic bacterium was isolated from the primary focus of infection or from a blood culture. Communications of the subarachnoid space with skin, sinuses, or mucosal surfaces were not considered distant foci of infection [27].

Appropriate antibiotic therapy was defined as administration of one or more antimicrobial agents shown to be effective against Gram-negative bacilli by susceptibility tests and capable of passing through the blood–brain barrier in an adequate amount, commenced either on the day of admission or before the deterioration of neurological and systemic conditions in inpatients [11,15].

The findings of impaired mental status, seizures and focal neurological signs detected on admission or subsequently, were considered neurological complications of bacterial meningitis. The development of septic shock, acute respiratory failure, acute renal failure, or consumption coagulopathy was considered a systemic complication if it was related to bacterial meningitis and was apparent on admission or shortly afterwards [27,28].

Nosocomial meningitis was defined as that developing more than 48 hours after admission or within one week after discharge [29].

Causes of death were classified in two categories: neurological causes (intractable seizures, brain herniation, cerebrovascular complications or coma) and systemic causes (septic shock, respiratory failure, disseminated intravascular coagulation or multiorgan dysfunction) [28].

### Statistical analysis

Qualitative variables were summarized with absolute numbers and percentage and quantitative variables with means and standard deviation (SD) or medians and interquartile range (IQR) (depending on their homogeneity).

Continuous variables were compared using t Student or the Mann–Whitney U test when appropriate. We analyzed categorical data using the χ^2^ test or Fisher’s exact test when indicated.

Logistic regression was used to determine characteristics of GNBM among patients with spontaneous acute bacterial meningitis. Additionally, we used logistic regression to examine the association between GNBM and the likelihood of death. Covariates were chosen based on previous research and clinical judgment. Proportions of the variation of the outcome explained by the model were assessed by the Nagelkerke’s R^2^.

All statistical tests were two-tailed, and a p value of <0.05 was considered to be statistically significant. Statistical analyses were performed using Statistical Product and Service Solutions (SPSS) software version 16 (SPSS Inc, Chicago, IL).

## Results

A total of 544 episodes of spontaneous acute bacterial meningitis in 529 patients were identified during the 25-year study period; 517 patients had single episodes of meningitis and 12 patients had more than one episode (nine had two episodes, and three patients had three episodes). Forty-eight percent of patients were male and the median age during the episodes of meningitis was 53 years (IQR 40). Etiology was established in 445 cases (81.8%). *Neisseria meningitidis* was the most common microorganism overall, accounting for 41.3% of the episodes; *Streptococcus pneumoniae* caused 28.8%, whereas *Listeria monocytogenes* and gram-negative bacilli other than *H. influenzae* caused 9.2% and 9.0%, respectively. Other pathogens were identified in 11.7%.

Of the 445 episodes of meningitis with known etiology, 40 (9.0%, 95% confidence interval [CI] 6.5-12.0%) were due to gram-negative bacilli.

### Characteristics of spontaneous Non-*H. influenzae* gram-negative bacillary meningitis (GNBM)

From January 1982 through June 1994, 21 episodes of GNBM from among 241 episodes of spontaneous acute meningitis (8.7%) were diagnosed and from July 1994 through December 2006, 19 cases from among 204 (9.3%) episodes (p = 0.82). The estimated overall annual incidence of GNBM was 2 cases per 100,000 adults.

By univariate analysis, patients with spontaneous GNBM were more likely than patients with other meningitis to be older, to have underlying diseases and to have a Charlson comorbidity index ≥3 (Table 1). Episodes of GNBM were also significantly more likely to be hospital acquired than were episodes of non-GNBM. The classic meningeal triad of fever, neck stiffness and altered mental status, as well as headache, and rash were less frequent among patients with GNBM than among patients with non-GNBM. Patients with GNBM also had higher overall rates of neurological complications – particularly impaired mental status -, systemic complications - specifically septic shock, acute respiratory failure, and acute kidney injury -, and death than patients with other causes of meningitis.

**Table 1:** Characteristics of episodes of spontaneous meningitis due to gram-negative bacilli and those due to other pathogens*

**Characteristics**	**Gram-negative bacillary meningitis (n = 40)**	**Other causes of meningitis (n = 405)**	**P value**
Male sex	23/40 (57.5)	183/393 (46.6)	0.187
Age – years, median (IQR)	64 (17)	50 (41)	< 0.001
Comorbid conditions	29/40 (72.5)	173/403 (42.9)	<0.001
• Cancer	8/40 (20.0)	41/403 (10.2)	0.067
• Diabetes mellitus	7/40 (17.5)	45/403 (11.2)	0.298
• Alcoholism	6/40 (15.0)	43/403 (10.7)	0.425
• Liver cirrhosis	4/40 (10.0)	17/403 (4.2)	0.110
Charlson comorbidity index ≥ 3	10/39 (25.6)	50/399 (12.5)	0.023
Distant focus of infection	31/40 (77.5)	139/402 (34.6)	<0.001
• Urinary tract infection	13/40 (32.5)	2/405 (0.5)	<0.001
• Otitis or sinusitis	4/40 (10.0)	58/405 (14.3)	0.452
• Pneumonia	2/40 (5.0)	22/405 (5.4)	1.000
Route of acquisition			
• Hospital-acquired (vs. community-acquired)	9/40 (22.5)	8/405 (2)	<0.001
Symptoms on presentation			
• Fever	37/40 (92.5)	387/402 (96.3)	0.217
• Change in mental status	29/40 (72.5)	278/402 (69.2)	0.661
• Neck stiffness	24/40 (60.0)	330/400 (82.5)	0.001
• Triad of fever, neck stiffness, and change in mental status	16/40 (40.0)	231/401 (57.6)	0.032
• Headache	22/40 (55.0)	323/402 (80.3)	<0.001
• Nausea and/or vomiting	18/40 (45.0)	239/394 (60.7)	0.055
• Focal neurological deficits	12/40 (30.0)	74/402 (18.4)	0.093
• Coma	6/40 (15)	26/402 (6.5)	0.057
• Seizures	5/40 (12.5)	33/401 (8.2)	0.371
• Rash	1/40 (2.5)	148/402 (36.8)	<0.001
• Systolic blood pressure – mm Hg (SD)	122 (31)	126 (31)	0.507
• Diastolic blood pressure – mm Hg (SD)	69 (22)	75 (21)	0.087
CSF examination			
• White-cell count, median (IQR)	1650 (2886)	1120 (2865)	0.248
○ < 100/mm^3^	3/39 (7.7)	54/395 (13.7)	0.292
○ 100–999/mm^3^	9/39 (23.1)	132/395 (33.4)	0.188
○ > 999/mm^3^	27/39 (69.2)	209/395 (52.9)	0.051
• Protein – g/liter, median (IQR)	3.75 (4.32)	3.48 (5.03)	0.786
• CSF: blood glucose ratio, median (IQR)	0.13 (0.22)	0.20 (0.34)	0.448
Positive blood culture	27/40 (67.5)	218/401 (54.4)	0.111
Blood white-cell count, median (IQR)	17350 (13225)	16650 (10800)	0.258
Thrombocyte count- platelets/mm3, median (IQR)	175000 (173000)	185000 (125000)	0.196
Neurological complications	20/40 (50)	121/402 (30.1)	0.010
• Impaired mental status	16/40 (40)	70/401 (17.5)	0.001
• Seizures	9/40 (22.5)	50/403 (12.4)	0.073
• Focal neurological deficits	12/40 (30)	81/402 (20.1)	0.145
Systemic complications	24/40 (60)	123/395 (31.1)	<0.001
• Septic shock	15/40 (37.5)	63/402 (15.7)	0.001
• Disseminated intravascular coagulation	7/40 (17.5)	40/399 (10)	0.174
• Acute respiratory failure	15/40 (37.5)	56/402 (13.9)	<0.001
• Acute kidney injury	12/40 (30)	51/401 (12.7)	0.003
Outcome			
• Neurological sequelae	3/19 (15.8)	49/334 (14.7)	0.894
• In-hospital mortality	21/40 (52.5)	66/404 (16.3)	<0.001

CI = confidence interval; IQR = interquartile range; SD = standard deviation.

*Values are reported as no./no. evaluated (%), unless otherwise noted.

A multivariate model was established using the entire cohort to identify features independently associated with spontaneous GNBM. Characteristics associated with spontaneous GNBM by multivariate modeling included urinary tract infection as distant focus of infection (OR, 98.45; 95% CI, 16.17-599.29), nosocomial acquisition of infection (OR, 13.77; 95% CI, 3.58-53.01), cerebrospinal fluid white cell count ≥1,000/mm^3^ (OR, 4.93; 95% CI, 1.76-13.79), acute respiratory failure (OR, 4.3; 95% CI, 1.5-12.0), cancer (OR, 3.07; 95% CI, 1.06-8.89), advanced age (OR, 1.03, 95% CI, 1.002-1.106), absence of rash (OR, 0.03; 95% CI, 0.001-0.66), and hypotension at clinical presentation (OR, 0.03; 95% CI, 0.95-0.99) (Nagelkerke’s R^2^ = 0.53). When we only included variables existing at clinical presentation in the model, the same characteristics, except for acute respiratory failure, were associated with GNBM.

### Diagnosis and microbiology

Lumbar puncture was performed in all the patients and CSF showed at least a CSF finding suggestive of acute bacterial meningitis: an increased protein concentration in 34 cases (85%), a decreased ratio of CSF glucose to blood glucose in 35 cases (87%) and pleocytosis in 36 cases (90%). The Gram stain of CSF was positive in 15 patients (38%) and the culture was positive in 39 (97%). In one patient, CSF Gram stain and CSF culture revealed no microorganisms, but blood culture was positive.

*Escherichia coli* was the most common cause of spontaneous GNBM (15 episodes [38%]), followed by *Pseudomonas* species (10 episodes [25%]; 7 of them were *Pseudomonas aeruginosa*). Other causative pathogens were *Serratia* spp. and *Bacteroides* spp. (3 episodes [8%]), *Proteus mirabilis*, *Klebsiella* spp. and *Enterobacter cloacae* (2 episodes [5%]) and 1 episode (3%) caused by *Acinetobacter baumanii* and *Citrobacter freundii*; in one case a gram-negative anaerobic bacilli was identified, but genus and species could not be established. Coinfection with another pathogen was observed in 4 cases (10%) that were caused by *E. cloacae* and *Streptococcus viridans*, *Bacteroides intermedius* and *S. viridans*, *Bacteroides* spp. and *Peptostreptococcus* spp., and anaerobic Gram-negative bacilli and *Peptostreptococcus* spp., respectively. No resistance to third-generation cephalosporin was found among gram-negative bacilli causing GNBM.

### Treatment

Seventeen patients (43%) had received antibiotic therapy before the diagnosis of meningitis. Initial empirical antibiotic treatment was appropriate in 32 cases (80%). Twenty out of 39 (51%; data not available for 1 episode) patients received a single antibiotic class as primary medical treatment of their GNBM, including a betalactam antibiotic in 18 out of 20 cases, and chloramphenicol and an aminoglycoside in 1 episode each. Nineteen patients (49%) received combination antibiotic therapy that included a betalactam plus an aminoglycoside in all of them. Third and fourth generation cephalosporins were the betalactam most commonly used (21 out of 37 episodes [57%]), followed by carbapenems in 5 episodes; other betalactams were used in 11 cases.

Information about intrathecal therapy was available in 20 episodes. Amikacin was administered intrathecally in 9 cases and gentamicin in 1 case. Adjunctive steroids were administered to 7 patients (18%). The median duration of the antibiotic treatment was 18 days (range 1–42).

### Hospital-acquired versus community-acquired

Nine episodes (23%) of spontaneous GNBM were nosocomial. They were diagnosed 3, 5, 7, 15 and 23 days after hospital admission (this information was missing in 4 episodes). Table 2 compares characteristics of nosocomial and community-acquired spontaneous GNBM. Patients with nosocomial infection had fewer distant foci of infection (p = 0.016) and the meningeal triad (p = 0.061). Patients with nosocomial meningitis were more likely than patients with community-acquired infection to be caused by *P.aeruginosa* (p = 0.049) to have lower levels of diastolic blood pressure (p = 0.016) and less than 100 cerebrospinal fluid white cells per cubic millimeter (p = 0.009) at presentation. Additionally, they had neurological and systemic complications more often and a higher mortality rate, although these differences did not reach statistical significance.

**Table 2:** Characteristics of nosocomial and community-acquired episodes of spontaneous meningitis due to gram-negative bacilli*

**Characteristics**	**Hospital-acquired (n = 9)**	**Community-acquired (n = 31)**	**P value**
Male sex	3/9 (33.3)	20/31 (64.5)	0.134
Age – years, median (IQR)	61 (23)	65 (18)	0.486
Comorbid conditions	7/9 (77.8)	22/31 (71.0)	1.000
Charlson comorbidity index ≥ 3	1/9 (11.1)	9/30 (30.0)	0.400
Distant focus of infection	4/9 (44.4)	27/31 (87.1)	0.016
• Urinary tract infection	2/9 (22.2)	11/31 (35.5)	0.690
• Otitis or sinusitis	0/9 (0.0)	4/31 (12.9)	0.557
• Pneumonia	1/9 (11.1)	1/31 (3.2)	0.404
Microbiology			
• *Pseudomonas aeruginosa*	5/9 (55.6)	5/31 (16.1)	0.049
• *Escherichia coli*	2/9 (22.2)	13/31 (41.9)	0.494
Symptoms on presentation			
• Triad of fever, neck stiffness, and change in mental status	1/9 (11.1)	15/31 (48.4)	0.061
• Focal neurological deficits	3/9 (33.3)	9/31 (29.0)	1.000
• Seizures	3/9 (33.3)	2/31 (6.5)	0.065
• Systolic blood pressure – mm Hg (SD)	110 (58)	127 (30)	0.292
• Diastolic blood pressure – mm Hg (SD)	60 (38)	80 (19)	0.016
CSF examination			
• White-cell count, median (IQR)	2456 (4472)	1601 (2646)	0.689
○ < 100/mm^3^	3/9 (33.3)	0/30 (0.0)	0.009
○ 100–999/mm^3^	1/9 (11.1)	8/30 (26.7)	0.654
○ > 999/mm^3^	5/9 (55.6)	22/30 (73.3)	0.416
• Protein – g/liter, median (IQR)	4.66 (7.42)	3.70 (3.67)	0.567
• CSF: blood glucose ratio, median (IQR)	0.10 (0.51)	0.13 (0.22)	0.731
Positive blood culture	7/9 (77.8)	20/31 (64.5)	0.690
Neurological complications	6/9 (66.7)	14/31 (45.2)	0.451
Systemic complications	7/9 (77.8)	17/31 (55.8)	0.272
Outcome			
• In-hospital mortality	6/9 (66.7)	15/31 (48.4)	0.457

CI = confidence interval; IQR = interquartile range; SD = standard deviation.

*Values are reported as no./no. evaluated (%), unless otherwise noted.

### Outcome

The overall mortality rate for patients with spontaneous GNBM was 53% (21 out of 40 patients). Fifteen of these patients (37%) died because of neurological causes and 6 (15%) because of systemic complications. Thirteen out of 21 patients (62%) died between January 1982 and June 1994, and 8 out of 19 (42%) between July 1994 and December 2006 (p = 0.210). Ten percent of survivors had moderate-to-severe disability.

We establish a multivariate logistic regression model to measure the risk of mortality associated with GNBM adjusted for clinically important variables. Results are shown in Table 3. The risk of death was twenty times higher (OR 20.47; 95% CI, 4.03-103.93; p < 0.001) for patients with GNBM than for patients infected with meningococcal meningitis after adjustment for clinical predictors.

**Table 3:** **Risk of death associated with spontaneous non-*Haemophilus influenzae *gram-negative bacillary meningitis adjusted for other clinical predictors**

**Risk factor**	**Adjusted odds ratio (95% confidence interval)**	**p**
Etiology		
• *Neisseria meningitidis*	1.000 (reference)	
• Gram-negative bacilli	20.474 (4.034–103.929)	< 0.001
• *Listeria monocytogenes*	11.369 (2.174–59.441)	0.004
• *Streptococcus pneumoniae*	5.896 (1.485–23.407)	0.012
• Other microorganisms	5.673 (1.096–39.379)	0.039
Age (≥ 65 years)	3.373 (1.462–7.780)	0.004
Positive blood culture	2.818 (1.079–7.359)	0.034
Inappropriate initial antibiotic therapy	13.184 (3.520–49.378)	< 0.001
Shock	8.929 (3.037–26.258)	< 0.001
Coagulation disorder	4.243 (1.149–15.666)	0.030
Acute renal failure	3.884 (1.440–10.473)	0.007
Neurological complications	19.441 (7.782–48.566)	< 0.001

Nagelkerke’s R^2^ of the adjusted model = 0.710.

## Discussion

Three patterns of meningitis caused by gram-negative bacilli have been described: neonatal, secondary to trauma or neurosurgery, and spontaneous in adults [12].

Most of the later series of GNBM have included both traumatic (secondary to head/spinal trauma or neurosurgical procedures) and spontaneous forms and have demonstrated that traumatic forms have specific characteristics that conclusively differentiate them from spontaneous meningitis [11,12,14-16,30-32]. Moreover, no previous studies have compared cases of spontaneous GNBM with cases of spontaneous meningitis due to other microorganisms. Using a large, prospectively collected single-hospital cohort of more than 500 patients with spontaneous meningitis, we document the characteristics of spontaneous gram-negative bacillary meningitis in adults and demonstrate that spontaneous meningitis due to gram-negative bacilli exhibits distinct characteristics as compared with spontaneous meningitis due to other pathogens.

Since most of the previous reports have combined traumatic and spontaneous forms, the true incidence of spontaneous meningitis is not well known [19]. Our study shows that gram-negative bacilli are a significant cause of spontaneous meningitis, accounting for 9% of the spontaneous meningitis of known etiology in adults; indeed, gram-negative bacilli would currently be the third or fourth leading cause of nontraumatic meningitis in adults following *S. pneumoniae* and *N. meningitidis*, and with a similar percentage to *L. monocytogenes* (9.2% in this serie).

Several studies have pointed to an increase in the frequency of GNBM over the last decades [11-13,21,31,33]. This increase would have occurred at the expense of a greater incidence of post-neurosurgical forms that may be explained by the significant rise of head and spinal cord surgical procedures in recent decades [32]. However, we did not find changes in the percentage of spontaneous GNBM during the span of the study.

*E. coli* was the most common cause of spontaneous GNBM in our study (almost 40%), as in most of the previous series in Europe and the United States [18-20]. In Taiwan and other countries in Southeast Asia, *K. pneumoniae* is often the most common pathogen of spontaneous GNBM, with percentages as high as 70% whereas *Klebsiella* spp. was an infrequent cause of spontaneous gram-negative bacillary meningitis (5%) in this and prior reports in Europe and the United States [1,7,12,15,19,32]. Not surprisingly, we found that causative microorganisms were different in nosocomial compared to community-acquired non-traumatic GNBM. Thus, while *E. coli* was the most common cause of community-acquired infections, *Pseudomonas* was the leading cause of nosocomial infections. We did not find gram-negative bacilli resistant to third-generation cephalosporins as etiologic agents of spontaneous GNBM in adults.

Spontaneous GNBM has been described as occurring most frequently in patients who are elderly and have severe underlying conditions, such as alcoholism, cirrhosis, diabetes, malignancy and other causes of immunosuppression. In our study, multivariate analysis demonstrated that patients with spontaneous GNBM were older than those with spontaneous meningitis due to other microorganisms. Additionally, 73% of patients with GNBM had co-morbid conditions, a percentage consistent with previous reports in which 60-80% of adults with non-traumatic meningitis due to gram-negative bacilli had underlying diseases. We found that cancer was the most common co-morbid condition, in contrast to older series that showed alcoholism, cirrhosis and diabetes as the most frequent underlying conditions [11,12,14-16,18-20,30,31].

In agreement with previous studies, we found that non-traumatic GNBM was more commonly a community–acquired infection. However, the risk for nosocomial acquisition of spontaneous meningitis due to gram-negative bacilli was already recognized in the earlier studies. Indeed, hospital-acquired infections have accounted for a substantial percentage of GNBM in previous reports, ranging from 20% to 38% (23% in our series) [12,18,20]. Moreover, in the current study, the multivariate model showed that patients with spontaneous GNBM were more likely to have acquired their infection in the hospital than patients with spontaneous meningitis due to other pathogens. Prior reports have underlined that most nosocomial meningitis are related to neurosurgical procedures or head trauma, and spontaneous hospital-acquired meningitis are considered to be very uncommon [10,21,27,34-36]. Although no conclusion can be drawn on hospital-acquired spontaneous meningitis, based upon our results, gram-negative bacilli can probably be considered the most frequent cause, and therefore empiric therapy of patients who develop spontaneous meningitis in hospital must cover gram-negative bacilli, including *Pseudomonas*.

It is thought that GNBM occurs most often as a complication of bacteremia from a distant focus of infection [12,20,37]. We saw that patients with non-traumatic meningitis due to gram-negative bacilli (and predominantly those with a community-acquired infection) had a distant source of infection more often than patients with spontaneous meningitis due to other pathogens. Specifically, multivariate analysis identified urinary tract as focus of infection as the most important independent factor associated with a higher risk of spontaneous gram-negative bacilli meningitis. This finding is clinically relevant, since a gram-negative bacillary etiology should be suspected in adults with spontaneous acute meningitis who have a history or a simultaneous diagnosis of bacteremic urinary tract infection. Moreover, given the great increase in elderly patients with underlying diseases such as malignancies, and the high prevalence of urinary infections in the community, it may happen that spontaneous GNBM may become a more common diagnosis or that it is currently underdiagnosed. The possibility of underdiagnosis could be facilitated by its clinical presentation with less frequent classic signs and symptoms of meningeal infection (such as the classic meningeal triad or headache). Consequently, the clinical picture would be easily explained by a urinary sepsis whereas the diagnosis of meningitis would be missed.

Patients with non-traumatic GNBM had more severe disease than patients with spontaneous meningitis due to other microorganisms, as reflected by a higher frequency of systemic and neurological complications and a higher mortality rate. Additionally, a previous study identified three baseline clinical features – hypotension, altered mental status, and seizures - as independently associated with an adverse outcome (defined as in-hospital dead or neurological deficit at discharge) [38]. Our multivariate analysis showed that hypotension at clinical presentation was associated with a dismal outcome, pointing to a greater systemic compromise.

The high mortality rate in this cohort (53%) is consistent with that in previous reports of spontaneous GNBM in adults, which has ranged from 40% to 60% [11,12,19,20]. In agreement with previous studies, we found that advanced age (≥65 years), positive blood cultures, inappropriate initial antibiotic therapy, and neurological and systemic complications (shock, coagulation disorder and acute renal failure) were associated with greater mortality in patients with spontaneous bacterial meningitis [11,12,19,29]. Additionally, we demonstrated that patients with spontaneous GNBM were at the highest risk of death among patients with spontaneous bacterial meningitis, after adjusting for clinical predictors. On the other hand, gram-negative bacilli as a cause of spontaneous meningitis were the strongest predictor of mortality in patients with non-traumatic acute bacterial meningitis. Previous reports have shown that patients with traumatic forms of GNBM have a better outcome than patients with spontaneous forms of infection; these studies found than patients with non-traumatic GNBM were older and had co-morbid conditions more often [11,12,14,16,31]. However, the role of the intrinsic virulence of gram-negative bacilli cannot be excluded. Since overall mortality is analyzed in the current investigation, gram-negative bacilli as causative agents of spontaneous meningitis probably represent a composite of prognostic factors including characteristics related to the virulence of causative organisms and, most importantly, host factors.

Our study has inherent limitations. First, although our study includes a large cohort of patients with spontaneous acute bacterial meningitis and is one of the largest series of spontaneous gram-negative bacillary meningitis, the number of cases of meningitis due to gram-negative bacilli is relatively small and there may be other factors associated with this infection that we were not able to identify in our model. Second, we analyzed overall in-hospital mortality and causes of death not directly related to meningitis could have been included. Finally, the end point of mortality was assessed at hospital discharge, and it therefore does not reflect meningitis outcome at a specific time after diagnosis.

## Conclusion

In summary, gram-negative bacilli currently rank third or fourth as the cause of spontaneous acute bacterial meningitis in adults (following *S. pneumoniae* and *N. meningitidis* or *S. pneumoniae, N. meningitidis* and *L. monocytogenes*), and the percentage of gram-negative bacillary meningitis among cases of spontaneous meningitis has not changed in the last decades. Patients with non-traumatic GNBM compared with patients with spontaneous meningitis due to other pathogens are older and more often have a history of cancer, a urinary tract infection as distant focus of infection, a nosocomial acquisition of infection, and a more severe disease with greater systemic compromise and a higher mortality rate. The incidence of spontaneous GNBM may increase in the future in association with an aging population with comorbidities such as malignancies, and the raised frequency of gram-negative sepsis. Additionally, because of the high associated mortality rate and the risk of underdiagnoses, clinicians should be alert to the possibility of meningitis in older patients with underlying diseases and urinary tract infections who develop fever and change in mental status.

## Competing interests

The authors declare that they have no competing interests.

## Authors’ contributions

VP, PD detected the patients, conceived of the study, participated in its design and wrote the first draft of the manuscript. NB contributed to the analysis, interpretation of the results and critical revisions of the manuscript. JLC, MG, and PC contributed to the interpretation of the results and critical revisions of the manuscript. All authors read and approved the final manuscript.

## Pre-publication history

The pre-publication history for this paper can be accessed here:

http://www.biomedcentral.com/1471-2334/13/451/prepub
